# Molecular Characteristics of *Klebsiella pneumoniae* Isolates From Outpatients in Sentinel Hospitals, Beijing, China, 2010–2019

**DOI:** 10.3389/fcimb.2020.00085

**Published:** 2020-02-28

**Authors:** Bing Lu, Changying Lin, Haican Liu, Xin Zhang, Yi Tian, Ying Huang, Hanqiu Yan, Mei Qu, Lei Jia, Quanyi Wang

**Affiliations:** ^1^Institute for Infectious Disease and Endemic Disease Control, Beijing Center for Disease Prevention and Control, Beijing Research Center for Preventive Medicine, Beijing Key Laboratory of Diagnostic and Traceability Technologies for Food Poisoning, Beijing, China; ^2^State Key Laboratory for Infectious Disease Prevention and Control, Collaborative Innovation Center for Diagnosis and Treatment of Infectious Diseases, National Institute for Communicable Disease Control and Prevention, Chinese Center for Disease Control and Prevention, Beijing, China

**Keywords:** *Klebsiella pneumoniae*, multi-locus sequencing type (MLST), whole genome sequencing (WGS), molecular typing, ESBL

## Abstract

**Background:** *Klebsiella pneumoniae* is an opportunistic pathogen associated with community-acquired and nosocomial infections. Since 2010, *K. pneumoniae* testing has been included into an existing diarrhea-syndrome surveillance system for estimating the prevalence of *K. pneumoniae* in diarrhea-syndrome patients, assessing antibiotic susceptibility, and investigating molecular characteristics of *K. pneumoniae*.

**Methods:** *Klebsiella pneumoniae* strains were isolated from stool specimens from diarrhea-syndrome outpatients in Beijing, China. Isolates were tested for antibiotic susceptibility, and phylogenetic relationships were explored though whole genome sequence analysis. Multi-locus sequence type (MLST) alleles were extracted from the whole genome sequence (WGS) data. A maximum likelihood tree was generated by MEGAX. Genomes were annotated by Prokka; core genes were produced by Roary; a maximum likelihood phylogenetic tree was generated using FastTree.

**Results:** Forty-four *K. pnuemoniae* strains were isolated from 2010 to July 2019; of these 37 were *K. pneumoniae* and seven were *K. variicola*. Antibiotic susceptibility testing showed that all 44 strains were sensitive to gentamicin, imipenem, amikacin, meropenem, kanamycin; 97.73% were sensitive to cefoxitin andlavo-ofloxacin; the highest antibiotic resistance rate was 79.55%, which was to ampicillin. We found three extended-spectrum beta-lactamase (ESBL) producing strains; we identified high-virulence ST types, including ST307 and ST65; and we found that ST23 has been the epidemic clone since 2010. MLST and core genome sequence analysis showed two distinct clusters of 44 *K. pnuemoniae*; 40 alleles were identified in core genome sequence analysis, while 36 alleles were identified in MLST typing.

**Conclusions:** There is an urgent need for epidemiological and molecular studies to understand the dynamics of antibiotic resistance and virulence gene transmission to guide strategies for *K. pneumoniae* surveillance. WGS analysis provided high discrimination power and reliable and robust data useful for molecular epidemiology.

## Background

*Klebsiella pneumoniae* is ubiquitous in the environment. *K. pneumoniae* is a Gram-negative opportunistic pathogen associated with community-acquired and nosocomial infections (Moradigaravand et al., 2017). Clinically, *K. pneumoniae* causes pneumoniae, abscesses, bacteremia, urinary tract infections (Podschun and Ullmann, 1998; Wyres and Holt, 2016), and occasionally, diarrhea (Moradigaravand et al., 2017). Nosocomial infections caused by *K. pneumoniae* impose an increasing risk of community infection.

Since 2010, *K. pneumoniae* testing has been included in an existing enteric pathogen surveillance system focused on diarrhea-syndrome outpatients of all ages in 245 sentinel hospitals of the 16 districts of Beijing (Lu et al., 2017). The aim of the system is to monitor the prevalence of *K. pneumoniae* in diarrhea-syndrome outpatients, assess antimicrobial resistance, and explore molecular characteristics of community-acquired *K. pneumoniae* infection strains.

## Methods

### Identification of Bacterial Strains

From 2010 to July 2019, stool specimens collected from diarrhea-syndrome outpatients in sentinel hospitals were analyzed using a reverse transcription polymerase chain reaction (RT-PCR) for diarrhea-generating viruses (e.g., rotavirus, norovirus, and calicivirus) (Deng et al., 2012; Gao et al., 2012; Ying et al., 2017) and cultured for isolation of diarrhea-generating bacteria. Any isolated bacteria strains were further tested to identify the pathogens (e.g., *Salmonella, Shigella, Escherichia coli, Vibrio parahemolyticus*, or *K. pneumoniae*) usingVitek2 Compact Instrument (bioMérieux; Marcy, France).

Isolated *K. pneumoniae* strains were tested for antibiotic susceptibility, deoxyribonucleic acid (DNA) extraction, whole-genome sequencing (WGS) analysis, and determination of their molecular characteristics.

### Antimicrobial Resistance Testing

Antimicrobial resistance testing for *K. pneumoniae* strains was assessed using the minimal inhibitory concentration (MIC) method. MICs were interpreted in accordance with the Clinical and Laboratory Standards Institute (CLSI) document, M100-S29:2019. Twenty-seven antimicrobials obtained from Shanghai Xingbai Co. (AST Panel for Aerobic Gram Negative bacilli) were used for antimicrobial resistance testing: ampicillin, ampicillin-sulbactam, amoxicillin with clavulanate potassium, cephazoline, cefepime, cefotaxime, cefoxitin, ceftazidime, aztreonam, imipenem, meropenem, gentamicin, amikacin, kanamycin, azithromycin, tetracycline, minocycline, doxycycline, nalidixic acid, ciprofloxacin, lavofloxacin, gemifloxacin, trimethoprim-sulphamethoxazole, sulfisoxazole, chloramphenicol, cefotaxime with clavulanate, and ceftazidime with clavulanate. *Escherichia coli* ATCC 25922 was used as a quality-control strain. MIC levels at 2 μg/mL or above for cefotaxime indicated a possible extended-spectrum beta-lactamase (ESBLs)-producing strain, requiring further confirmation. MIC for ceftazidime combined with clavulanatede creasing at least three two-fold concentrations compared with the MIC value for ceftazidime alone (e.g., ceftazidime MIC = 8 μg/mL; ceftazidime-clavulanate MIC = 1 μg/mL) confirmed an ESBL-producing strain.

### DNA Extraction and WGS

DNA was extracted by QIAamp DNA Mini Kit (Qiagen, Hilden, Germany). Quantification of extracted genomic DNA (gDNA) was determined on a NanoDrop spectrophotometer, with verification by agarose gel electrophoresis and fluorometric analysis (Qubit2.0).

Multiplexed paired-end libraries (2 × 300 bp) were prepared for DNA sequencing using the NEBNext®Ultra™ DNA Library Prep Kit for Illumina (NEB, USA). Sequences were determined on an Illumina PE150 platform with 100 × coverage at Beijing Novogene technology Co., Ltd.

Raw sequencing data were checked for quality, trimmed, and assembled *de novo* into contiguous segments using CLC Genomics Workbench version 10.1.1 (CLC, Bio-QIAGEN, Aarhus, Denmark) and SPAdes version 3.13 (Bankevich et al., 2012).

The WGS data were matched in the NCBI BLAST database to identify three distinct species of *K. pneumoniae*: *K. pneumoniae* (KpI), *K. quasipneumoniae* (KpII), and *K. variicola* (KpIII) (Holt et al., 2015).

### Plasmid, Antimicrobial Resistant Genes and Multi-Locus Sequence Type (MLST) Analysis

The genomic analysis was based on the Center for Genomic Epidemiology web server (https://cge.cbs.dtu.dk/services/cge/), in which web-based multi-locus sequence type (MLST) 2.0 (Larsen et al., 2012), ResFinder 3.2 (Zankari et al., 2012), and PlasmidFinder 2.1 (Carattoli et al., 2014) were used for cluster sequencing types, investigating antimicrobial resistant genes, and defining content of plasmid replicon types, respectively.

MLST analyses were performed using seven housekeeping genes (*gapA, infB, mdh, pgi, phoE, rpob*, and *tonB*) to characterize diversity and epidemiology of *K. pneumoniae* isolates (Diancourt et al., 2005). WGS data were used to generate MLST assignments for each isolate; unknown STs were sent to the *Klebsiella pneumoniae* MLST database at the Pasteur Institute (https://bigsdb.pasteur.fr/klebsiella/klebsiella.html). Genotyping analysis was based on MLST sequences; maximum likelihood trees were generated by MEGA-X (Kumar et al., 2018).

### Annotation and Core Genome Analysis

Genomes were annotated by Prokka, a tool for rapid prokaryotic genome annotation (Seemann, 2014). Phylogenetic analyses were produced by Roary, a tool that rapidly builds large-scale pan genomes and identifies core genes (shared by all strains) and accessory genes (Page et al., 2015). A maximum likelihood phylogenetic tree was generated by FastTree version 2.1.10 (Price et al., 2010) to assess relatedness among genomes in the isolated bacteria and to approximate the species tree.

## Results

Surveillance led to isolation of 1 to 11 *K. pneumoniae* strains each year, identifying 44 *K. pneumoniae* strains from 25,411 stool specimens in 10 years, the detection rate was 0.17% (44/25,411).

### Antimicrobial Resistance

All 44 *K. pneumoniae* strains were sensitive to gentamicin, imipenem, amikacin, meropenem, kanamycin; 97.7% were sensitive to cefoxitin andlavo-ofloxacin; 95.5% were sensitive to nalidixic acid, azithromycin, and ciprofloxacin; 79.6% of isolated *K. pnuemoniae* strains manifested resistance to ampicillin, and 13.6% of isolated staring showed resistance to sulfisoxazole, trimethoprim, and sulphame-thoxazole (Table 1). Three *K. pneumoniae* strains were confirmed as ESBL-producing strains (Table 2).

**Table 1:** Antibiotic susceptibility results for 44 *K. pneumoniae* strains.

**Antibiotic**		**Resistant *n***	**Intermediate *n***	**Susceptible *n***
Penicilins	Ampicillin	35, 79.55%	7,15.91%	2, 4.55%
β-Lactam/β-lactamase inhibitor combinations	Amoxicillin with clavulanate potassium	2, 4.55%	3,4.55%	40, 90.91%
	Ampicillin-sulbactam	4, 9.09%	2, 4.55%	28, 86.36%
Cephems	Cephazoline	4, 9.09%	3, 6.82%	37, 84.09%
	Cefepime	3, 6.82%	0, 0	41, 93.18%
	Cefotaxime	4, 9.09%	0, 0	40, 90.91%
	Cefoxitin	1, 2.27%	0, 0	43, 97.73%
	Ceftazidime	0, 0	3, 6.82%	41, 93.18%
Monobactams	Aztreonam	3, 6.82%	0, 0	41, 93.18%
Carbapenems	Imipenem	0, 0	0, 0	44,100.00%
	Meropenem	0, 0	0, 0	44,100.00%
Aminoglycosides	Gentamicin	0, 0	0, 0	44,100.00%
	Amikacin	0, 0	0, 0	44,100.00%
	Kanamycin	0, 0	0, 0	44,100.00%
Macrolides	Azithromycin	2, 4.55%	0, 0	42, 95.45%
Tetracyclines	Tetracycline	5, 11.36%	2, 4.55%	37, 84.09%
	Minocycline	2, 4.55%	13, 29.55%	29, 65.91%
	Doxycycline	3, 6.82%	4, 9.09%	37, 84.09%
Quinolons and fluoroquinolones	Nalidixic acid	2, 4.55%	0, 0	42, 95.45%
	Ciprofloxacin	1, 2.27%	1, 2.27%	42, 95.45%
	Lavo-floxacin	1, 2.27%	0, 0	43, 97.73%
	Gemifloxacin	2, 4.55%	1, 2.27%	41, 93.18%
Folate pathway inhibitors	Trimethoprim-sulphamethoxazole	6, 13.64%	0, 0	38, 86.36%
	Sulfisoxazole	6, 13.64%	0, 0	38, 86.36%
Phenicols	Chloramphenicol	4, 9.09%	1, 2.27%	39, 88.64%

**Table 2:** Summary of species, and genotypic characteristics for 44 *K. pneumoniae* strains.

**Strain ID**	**Isolation year**	**Species**	**MLST type**	**Plasmid replicon type**	**ESBL strain**	**Antibiotic resistance genes**									
						**Aminoglycoside**	**Beta-lactam**	**Quinolone**	**Fosfomycin**	**Phenicol**	**Sulphonamide**	**Tetracycline**	**Trimethoprim**	**Macrolide**	**Rifampicin**
BJ2010005-S1	2010	*Klebsiella variicola*	ST2362	ColRNAI			blaLEN13	oqxA,oqxB	fosA						
BJ2011355-S2	2011	*Klebsiella variicola*	ST197				blaLEN16	oqxA,oqxB	fosA						
BJ2011367-S32	2011	*Klebsiella pneumoniae*	ST23				blaSHV-36	oqxA,oqxB	fosA						
BJ2011375-S36	2011	*Klebsiella pneumoniae*	ST2363	ColRNAI, ColpVC			blaSHV-1	oqxA,oqxB	fosA						
BJ2012015-S3	2012	*Klebsiella pneumoniae*	ST23				blaSHV-36	oqxA,oqxB	fosA						
BJ2012035-S4	2012	*Klebsiella pneumoniae*	ST2364				blaSHV-11	oqxA,oqxB	fosA						
BJ2012036-S33	2012	*Klebsiella pneumoniae*	ST218				blaSHV-1	oqxA,oqxB	fosA						
BJ2013059-S5	2013	*Klebsiella pneumoniae*	ST20	ColRNAI			blaSHV-83		fosA						
BJ2013082-S34	2013	*Klebsiella pneumoniae*	ST412				blaSHV-11	oqxA,oqxB	fosA						
BJ2013086-S7	2013	*Klebsiella pneumoniae*	ST1660				blaSHV-36	oqxA,oqxB	fosA						
BJ2013261-S8	2013	*Klebsiella pneumoniae*	ST1310	ColRNAI, Col(MGD2)			blaSHV-1	oqxA,oqxB	fosA						
BJ2014008-S21	2014	*Klebsiella pneumoniae*	ST23				blaSHV-36	oqxA,oqxB	fosA						
BJ2014021-S10	2014	*Klebsiella pneumoniae*	ST65				blaSHV-11	oqxA,oqxB	fosA						
BJ2014039-S57	2014	*Klebsiella pneumoniae*	ST2367	ColRNAI, Col(MGD2)			blaSHV-11	oqxA,oqxB	fosA						
BJ2014085-S12	2014	*Klebsiella pneumoniae*	ST592	ColRNAI			blaSHV-26	oqxA,oqxB	fosA						
BJ2014086-S13	2014	*Klebsiella pneumoniae*	ST34	ColRNAI			blaSHV-26	oqxA,oqxB	fosA						
BJ2014087-S14	2014	*Klebsiella pneumoniae*	ST2369	ColRNAI, Col(MGD2)			blaSHV-1	oqxA,oqxB	fosA						
BJ2014199-S15	2014	*Klebsiella pneumoniae*	ST17				blaSHV-11	oqxA,oqxB	fosA						
BJ2014201-S35	2014	*Klebsiella pneumoniae*	ST2370	ColRNAI, IncR		aadA2,aph(3″)-Ib	blaSHV-11	oqxA,oqxB	fosA	strA	sul1	tet(A)	dfrA12	mph(A)	
BJ2015035-S16	2015	*Klebsiella pneumoniae*	ST345				blaSHV-1	oqxA,oqxB	fosA						
BJ2016012-S17	2016	*Klebsiella pneumoniae*	ST485				blaSHV-27	oqxA,oqxB	fosA						
BJ2016022-S18	2016	*Klebsiella pneumoniae*	ST35	ColRNAI, Col(MGD2)			blaSHV-33	oqxA,oqxB	fosA						
BJ2017019-S19	2017	*Klebsiella variicola*	ST4448				blaLEN13	oqxA,oqxB	fosA						
BJ2017021-S20	2017	*Klebsiella pneumoniae*	ST23				blaSHV-36	oqxA,oqxB	fosA						
BJ2018022-S9	2018	*Klebsiella pneumoniae*	ST307	ColRNAI	Yes	aac(6′)lb-cr, aph(3″)-Ib, aph(6)-Id	blaCTX-M-15, blaSHV-28, blaOXA-1, blaTEM-1B	aac(6′)-Ib-cr,oqxA,oqxB,qnrB1	fosA		sul2	tet(A)	dfrA14		
BJ2018060-S11	2018	*Klebsiella variicola*	ST4447				blaLEN22	oqxA,oqxB	fosA						
BJ2018062-S23	2018	*Klebsiella pneumoniae*	ST1307	IncR, Col(MGD2)		aac(3)-IV, aac(6′)-Ib-cr, aadA1, aadA2, aph(4)-Ia	blaDHA-1, blaOXA-1, blaSHV-11	aac(6′)-Ib-cr, qnrB4	fosA	catB3,floR,cmlA1	sul1,sul2,sul3	tet(A)	dfrA12	mph(A)	ARR-3
BJ2018066-S24	2018	*Klebsiella pneumoniae*	ST4449				blaSHV-1	oqxA,oqxB	fosA						
BJ2018083-S25	2018	*Klebsiella pneumoniae*	ST3277				blaSHV-1	oqxA,oqxB	fosA						
BJ2018090-S26	2018	*Klebsiella variicola*	ST360				blaLEN13	oqxA,oqxB	fosA						
BJ2018100-S27	2018	*Klebsiella variicola*	ST4450				blaLEN24	oqxA,oqxB	fosA						
BJ2018102-S28	2018	*Klebsiella pneumoniae*	ST309				blaSHV-11	oqxA, oqxB, QnrS1	fosA	floR	sul2	tet(A)	dfrA14		
BJ2018103-S29	2018	*Klebsiella variicola*	ST4451				blaLEN24	oqxA,oqxB	fosA						
BJ2018104-S30	2018	*Klebsiella pneumoniae*	ST4452	ColRNAI	Yes		blaSHV-11, blaCTX-M-15, blaTEM-1B	oqxA,oqxB	fosA						
BJ2018114-S31	2018	*Klebsiella pneumoniae*	ST36	ColRNAI, IncR, Col(MGD2)			blaSHV-11	oqxA,oqxB	fosA						
BJ2019005-S58	2019	*Klebsiella pneumoniae*	ST564				blaSHV-11	oqxA,oqxB	fosA						
BJ2019024-S59	2019	*Klebsiella pneumoniae*	ST742				blaSHV-11	oqxA,oqxB	fosA						
BJ2019046-S95	2019	*Klebsiella pneumoniae*	ST101	ColRNAI,IncR			blaSHV-1	oqxA,oqxB	fosA				dfrA15		
BJ2019047-S96	2019	*Klebsiella pneumoniae*	ST39	ColRNAI,IncQ2			blaSHV-11	oqxA,oqxB	fosA						
BJ2019059-S97	2019	*Klebsiella pneumoniae*	ST412				blaSHV-1	oqxA,oqxB	fosA						
BJ2019060-S98	2019	*Klebsiella pneumoniae*	ST1537				blaSHV-1	oqxA,oqxB	fosA						
BJ2019061-S99	2019	*Klebsiella pneumoniae*	ST17	ColRNAI	Yes	aac(6′)-Ib-cr, aph(3″)-Ib, aph(6)-Id	blaCTX-M-15, blaOXA-1, blaTEM-1B, blaSHV-11	aac(6′)-Ib-cr,oqxA,oqxB,qnrB1	fosA	catB3	sul2	tet(A)	dfrA14		
BJ2019062-S100	2019	*Klebsiella pneumoniae*	ST23				blaSHV-36	oqxA,oqxB	fosA						
BJ2019070-S103	2019	*Klebsiella pneumoniae*	ST584	IncR			blaSHV-38	oqxA,oqxB	fosA						

### WGS NCBI Blast Results

Forty-four *K. pneumoniae* strains were disambiguated into two species: 37 K. pneumoniae (KpI) strains and 7 K. variicola (KpIII) strains. Surveillance did not identify any K. quasipneumoniae (KpII) strains (Table 2).

### MLST Results and MEGA Analysis

**Figure 1:**
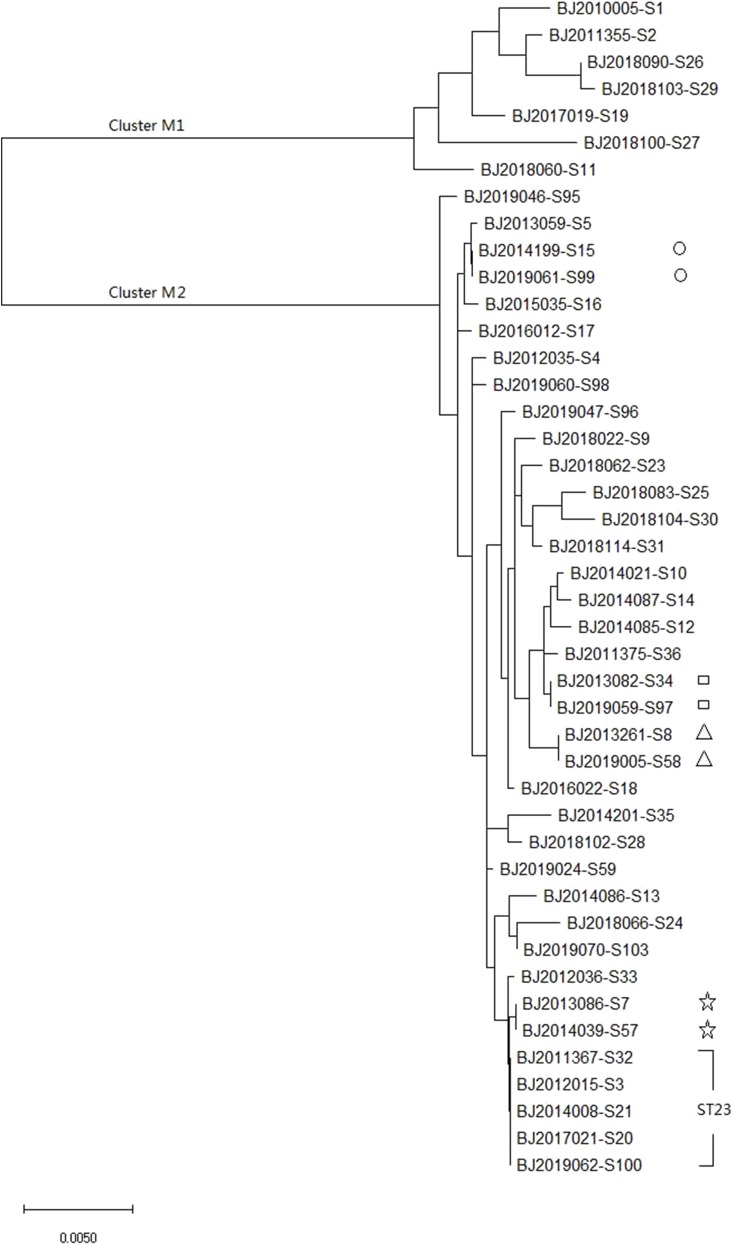
Molecular phylogenetic analysis by maximum likelihood method based on MLST sequences of 44 *K. pneumoniea* strains.

MLST of the 44 strains revealed 36 different sequence types (STs), including ST23, which has been detected five times in Beijing in the most recent 10 years, and ST4447, ST4448, ST4449, ST4450, ST4451, and ST4452, which were seen for the first time in the global database. The maximum likelihood tree identified 36 MLST alleles, and 44 strains were disambiguated into two clonal groups: Cluster M1 (containing 7 strains, *K. variicola* strains) and Cluster M2 (containing 37 strains, *K. pneumoniae* strains) (Figure 1).

### Plasmid and Drug Resistance Genes Identification

ColRNAI, Col(MGD2), ColpVC, IncR, and IncQ2 plasmid replicons were identified, encompassing 40.9, 13.6, 2.3, 11.4, and 2.3% of the 44 strains, respectively. Among the resistance genes to Beta-lactams, Quinolone, Fosfomycin, Phenicol, Sulphonamide, Tetracycline, Trimethoprim, Macrolide, and Rifampicin, resistance genesto Beta-lactam, Quinolone, and Fosfomycin were predominant. Among the resistance genes identified from 3 ESBL-producing strains, blaCTX-M-15 and blaTEM-1B were unique resistance genes to Beta-lactams (Table 2).

### Core Genome Analysis

**Figure 2:**
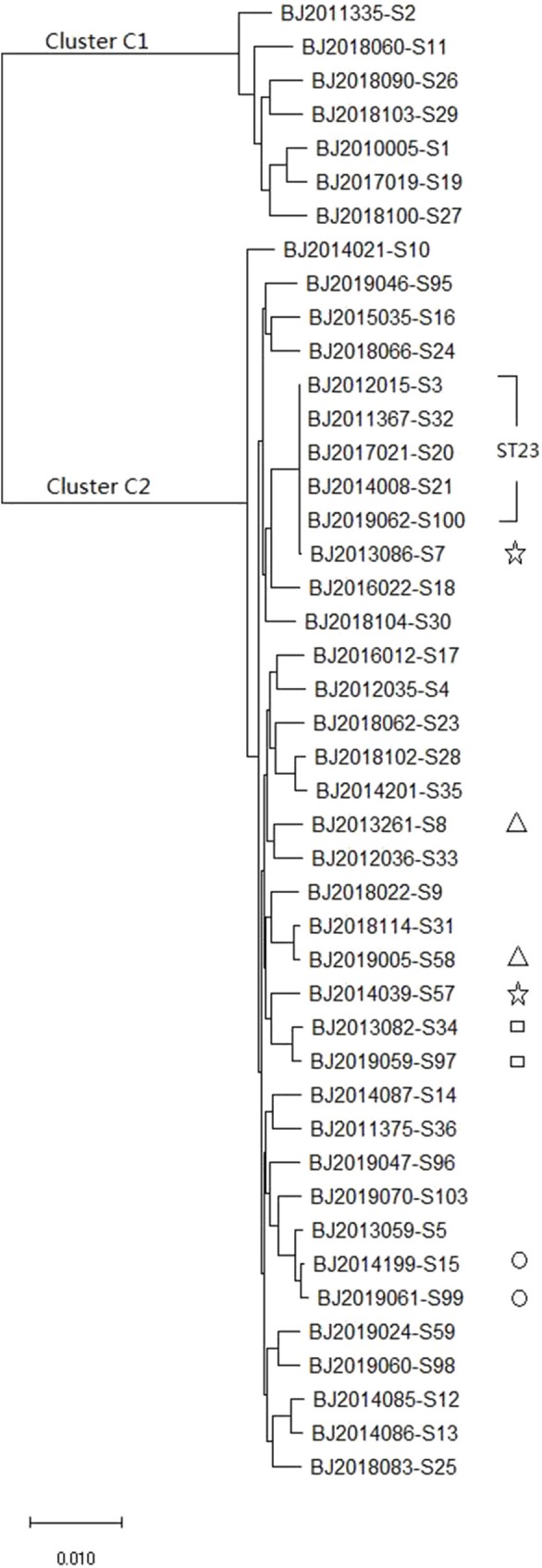
The maximum likelihood phylogenetic tree based on the core genome sequences of 44 *K. pneumoniae* strains.

The whole genome sequence of the 44 strains identified 3,428 core genes. In maximum-likelihood phylogenies trees, these core genome sequences showed 40 allele differences that grouped into two distinct clusters: cluster C1 (containing7 *K. varricola* strains), and cluster C2 (containing 37 *K. pneumoniae* strains) (Figure 2).

## Discussion

*K. pneumoniae* has been reported to be a leading cause of hospital associated infections and a common cause of community-acquired infections in many countries (Pendleton et al., 2013; Moradigaravand et al., 2017; Musicha et al., 2017). Beijing outpatient-based diarrhea-syndromes surveillance detected *K. pneumoniae* every year since 2010 demonstrating the existence of community-acquired infection caused by *K. pneumoniae*. Detection of five ST23 strains from 2010 to 2019 further demonstrated that the ST23 strain has persisted in Beijing throughout these years. Our results should alert public health officials since ST23 of *K. pneumoniae* has well-known virulence and is able to cause severe disease in otherwise healthy individuals (Turton et al., 2007; Brisse et al., 2009; Holt et al., 2015). It typically carries all four acquired siderophore systems as well as *rmpA* (Brisse et al., 2009). *K. pneumoniae* ST23 is the most predominant sequence type causing invasive community-acquired infections in Asia (Chung et al., 2012). Surveillance also detected an ST65 strain, which carries colibactin and *rmpA* (Brisse et al., 2009), and which is associated with lethal infections in humans and marine mammals (Liao et al., 2014).

The three community-acquired *K. pneumoniae* ESBL-producing strains (ST307, ST4452, and ST17) that were identified in the most recent 2-years period provide a significant signal of drug resistance in the population. All three ESBLs producing strains harbor blaCTX-M-15 and blaTEM-1B antibiotic resistance genes. CTX-M-15 belonged to the CTX-M-1 group, and is widespread in east Asia (Bonnet, 2004). The blaCTX-M was first reported in 1990 in a cefotaxime resistant *E. coli* strain isolated from the fecal flora of a laboratory dog (Bauernfeind et al., 1990). Since then, the CTX-M enzymes have formed a rapidly growing family of ESBLs distributed over wide geographic areas and among a wide range of clinical bacteria, particularly among members of the *Enterobacteriaceae* family (Bonnet, 2004). Outbreaks have been described in several countries (Yan et al., 2000; Baraniak et al., 2002). Since 1999, CTX-M has been reported to have become the most frequent ESBL in the Enterobacteriaceae in China (Chanawong et al., 2002; Xiong et al., 2002; Wang et al., 2003). Notably, the *K. pneumoniae* ST307 ESBL-producing strain has a novel lineage with potential to become an epidemic or “high-risk” clone. It has been recognized as a candidate for becoming one of the most clinically-relevant clones since its worldwide emergence during recent years (Villa et al., 2017). The ST307 lineage displays an association with CTX-M-15- and Carbapenemase (KPC)-producing encoding plasmids (Villa et al., 2017). The *K. pneumoniae* ST307 detected in our study did not harbor the blaKPC gene, but KPC producing factor could be acquired through horizontal plasmids transfer. The ability of this clone lineage to acquire novel genetic features may contribute to its increased persistence in the environment and highlights its potential public health threat of dramatically disseminated multiple drug resistance among bacteria.

MLST and core genomes sequences consistently differentiated 44 *K. pneumoniae* into 7 *K. varricola* strains and 37 *K. pneumoniae* strains. However, the core genome sequences increase discriminatory power for bacterial pathogen subtyping. For example, BJ2013086-S7 strain is very close to BJ2014039-S57 in an MLST molecular phylogenetic tree (see Figure 1), however, BJ2013086-S7 was separated from BJ2014039-S57in the phylogenetic tree generated by the core genome, and was closer to ST23 strain. Similar distinction was made for BJ2013261-S8 and BJ2019005-S58 stains, BJ2019059-S97 and BJ2013082-S34, and BJ2019061-S99 and BJ2014199-S15. Since WGS consists of sequencing chromosome information, both inherited from ancestors and their mutations, in theory, this powerful tool can deduce the chains of potential cross transmission of *K. pneumoniae* infection (Croucher and Didelot, 2015) and facilitate study of the population structure and pathogen evolution (Bialek-Davenet et al., 2014; Struve et al., 2015; Zhou et al., 2017). However, its discriminatory power relies on reliable, and robust, and long-term WGS data from different geographic areas. It will be valuable to establish a *K. pneumoniae* identification network for information sharing.

This study suffers two main limitations. First, the sentinel surveillance could have under-estimated the prevalence of *K. pneumoniae* in diarrhea-syndrome outpatients since *K. pneumoniae* in most of the circumstance is not the predominant causative-pathogen. Second, lack of comparation with molecular characteristics of hospital-acquired *K. pneumoniae* infection strains encourages more effort should be made to provide complete molecular spectrum in future studies.

## Conclusions

Outpatient-based diarrhea-syndrome surveillance in Beijing China identified 3 ESBLs-producing strains in 2018 and 2019 that had not been detected previously. We identified high virulence ST types, such as ST307 and ST65, and we showed that ST23 has been the epidemic clone since 2010. There is an urgent need for epidemiological and molecular studies to understand the dynamics of antibiotic resistance and virulence gene transmission to guide strategies for *K. pneumoniae* surveillance. WGS analysis provides high discrimination power, and reliable and robust data for molecular epidemiology.

## Data Availability Statement

The raw data supporting the conclusions of this article will be made available by the authors, without undue reservation, to any qualified researcher.

## Ethics Statement

The study was approved by the Ethics Committee of the Beijing Center for Disease Prevention and Control.

## Author Contributions

BL participated in data analysis and drafted the manuscript. CL and HL managed the bio information analysis. XZ and YH carried out the molecular genetic studies. HY participated in sample isolation. LJ and YT managed the strains and data collection. MQ and QW participated in the design of the study. All authors read and approved the final manuscript.

### Conflict of Interest

The authors declare that the research was conducted in the absence of any commercial or financial relationships that could be construed as a potential conflict of interest.

## Abbreviations

*K. pneumoniae*: *Klebsiella pneumonia*
*K. quasipneumoniae*: *Klebsiella quasipneumoniae*
*K. variicola*: *Klebsiella variicola*
MLST: multi-locus sequence typing
RT-PCR: reverse transcription polymerase chain reaction
DNA: deoxyribonucleic acid
WGS: whole genome sequence
CLSI: Clinical and Laboratory Standards Institute
ESBL: extended-spectrum beta-lactamase
MIC: minimal inhibitory concentration
STs: sequence types
KPC: Carbapenemase.
